# Design and development of orchard autonomous navigation spray system

**DOI:** 10.3389/fpls.2022.960686

**Published:** 2022-08-01

**Authors:** Shubo Wang, Jianli Song, Peng Qi, Changjian Yuan, Hecheng Wu, Lanting Zhang, Weihong Liu, Yajia Liu, Xiongkui He

**Affiliations:** Centre for Chemicals Application Technology, College of Science, College of Agricultural Unmanned System, China Agricultural University, Beijing, China

**Keywords:** orchard plant protection, crawler sprayer, autonomous navigation, laser lidar, obstacle avoidance

## Abstract

Driven by the demand for efficient plant protection in orchards, the autonomous navigation system for orchards is hereby designed and developed in this study. According to the three modules of unmanned system “perception-decision-control,” the environment perception and map construction strategy based on 3D lidar is constructed for the complex environment in orchards. At the same time, millimeter-wave radar is further selected for multi-source information fusion for the perception of obstacles. The extraction of orchard navigation lines is achieved by formulating a four-step extraction strategy according to the obtained lidar data. Finally, aiming at the control problem of plant protection machine, the ADRC control strategy is adopted to enhance the noise immunity of the system. Different working conditions are designed in the experimental section for testing the obstacle avoidance performance and navigation accuracy of the autonomous navigation sprayer. The experimental results show that the unmanned vehicle can identify the obstacle quickly and make an emergency stop and find a rather narrow feasible area when a moving person or a different thin column is used as an obstacle. Many experiments have shown a safe distance for obstacle avoidance about 0.5 m, which meets the obstacle avoidance requirements. In the navigation accuracy experiment, the average navigation error in both experiments is within 15 cm, satisfying the requirements for orchard spray operation. A set of spray test experiments are designed in the final experimental part to further verify the feasibility of the system developed by the institute, and the coverage rate of the leaves of the canopy is about 50%.

## Introduction

Among orchard management operations, plant protection management is provided with the highest labor intensity (Liu et al., 2020). Exploring an automatic sprayer with a high degree of automation and a high pesticide utilization is a hot spot for agricultural machinists. There are mainly two thorny problems in the development of automated orchard sprayers: one is to achieve efficient penetration of pesticides in a low dense canopy and reduce the loss of chemical solution (Meng et al., 2022; Wang C. et al., 2022), while the other is to let the machine traverse the orchard autonomously without manual control in the orchard with a closed canopy and blocked vision (Bergerman et al., 2015; Ye et al., 2018; Zhang et al., 2020). Considerable research has been conducted on the above two issues.

The leaves can rotate under the blowing of the centrifugal fan, and those of the fruit tree can be fully sprayed because of the air assistance of the air-driven sprayer (Boatwright et al., 2020; Boatwright and Schnabel, 2020). In general, the effect is better than that of hydraulic atomization, making the way of wind assistance more frequently adopted in the orchard. In the field of air-driven orchard sprayers, the development process proposed by the developed countries in Europe is relatively richer, where the development has always been committed to solving environmental problems such as low utilization of spray pesticides and pesticide pollution of soil and atmosphere (Fox et al., 2008; Boatwright and Schnabel, 2020). The porous air bag orchard spray, the multi-airway orchard spray and the small orchard air-driven sprayer have been developed consecutively (Fox et al., 2008). At this stage, the planting mode of orchards has been standardized and transformed accordingly, which has promoted the development and application of air-assisted sprayers in small and medium-sized orchards, making it a main force of orchard spray plant protection management machinery under the dwarf dense planting mode (Owen-Smith et al., 2019; An et al., 2020).

Another thorny problem of orchard spray is to realize automatic driving. In the complex and closed environment of the orchard, many sensors and positioning devices may be subject to a low accuracy or even failure (Fei and Vougioukas, 2022). Exploring a reliable autonomous driving scheme in orchard has been extensively studied. After decades of exploration, a complete unmanned system scheme with “perception-decision-making-control” progression has been formed (Bergerman et al., 2015; Jones et al., 2019). At the level of “perception,” sensors are mainly used to obtain orchard environmental data, including fruit tree information, road information, etc., which will be then applied to make the next-stage decision; at the level of “decision making,” the obtained environmental information is further processed, and then the travel track is extracted for judging whether there are obstacles; and at the level of “control,” the controller is designed to drive the system to follow the trajectory. The result of this research shows that in the entire autonomous driving process, it is the most difficult task to effectively perceive the orchard environment, perform real-time positioning, and effectively avoid obstacles. Visual sensors or radar sensors are generally used as the perception part, the representative literature, and the advantages and disadvantages are listed in Table 1.

**TABLE 1:** Classification of driverless perception and decision system working in orchard.

	Advantage	Shortcoming
GNSS (Guevara et al., 2020; Mao et al., 2022)	It can work in the orchard all day and is completely unaffected by the weather	In the orchard, the loss of signal caused by canopy occlusion, multipath effect, radio frequency interference, etc., results in great errors to GNSS navigation and even led to invalid navigation
Binocular vision (Stefas et al., 2016; Lin et al., 2021; Ma et al., 2021; Vrochidou et al., 2022)	Low cost and abundant information (depth map and RGB map)	The accuracy is poor, and is seriously reduced in dim light and at night, failing to meet the needs of overnight operation in orchards
Lidar (Bergerman et al., 2015; Blok et al., 2019; Jones et al., 2019; Guevara et al., 2020; Zhang et al., 2020)	The cost is high, and is greatly affected by bad weather such as rain and snow	The cost is high, and is greatly affected by bad weather such as rain and snow
Millimeter wave radar (Li X. et al., 2020; Wang et al., 2021)	It has a strong penetrability and is not affected by light, and can meet all kinds of weather in the orchard	The atmospheric attenuation is large and the detection distance is short, so it cannot be perceived in a large range

In general, GNSS-based orchard navigation and positioning is based on satellite map or known structured orchard map, which fails to realize obstacle avoidance and navigation in unknown environment. Millimeter wave radar can only sense obstacles, but it cannot build a global map. Therefore, the above two methods are generally combined with other sensors to guide the unmanned system through the fusion strategy. Therefore, the development of autonomous obstacle avoidance and navigation orchard vehicles based on binocular vision, lidar, or multi-sensor fusion has been extensively studied. A previous study (Chen et al., 2021) adopted a binocular vision method to build a simultaneous localization and mapping (SLAM), which realized the perception of orchard environment through vision and generated a detailed global map supporting long-term, flexible, and large-scale orchard picking. On the basis of binocular vision, the study discussed in Liu et al. (2022) proposed a trinocular vision system for orchard vehicle based on a wide-angle camera and binocular stereo vision system, which finally realized orchard row detection and obstacle detection simultaneously. Besides, based on the vision technology, the study discussed in Li Y. et al. (2020) proposed a visual perception method based on convolutional neural network, and realized obstacle detection and colligation avoidance in robot harvesters. Ravankar et al. (2021) used lidar sensors to navigate unmanned vehicles in the vineyard, and developed a point cloud processing algorithm to avoid dynamic obstacles in the vineyard while smoothing the robot’s trajectories. A previous study (Ji et al., 2021) built a tracker platform based on 3D/2D lidar and GNSS/AHRS to acquire fusion point cloud data, and finally realized obstacle perception and target tracking. The study explained in Kragh and Underwood (2020) adopted the fusion of lidar and visual sensors and proposed a multimodal fusion algorithm from the scene analysis domain for obstacle detection in agriculture with moving ground vehicles. One of the previous studies (Emmi et al., 2021) proposed the field autonomous navigation system based on 2D lidar and RGB cameras, and realized the robot positioning in a hybrid topological map through data fusion. Previous research (Rovira-Más et al., 2020) proposed an autonomous navigation strategy based on the integration of three sensing devices, namely, 3D vision, lidar, and ultrasonics. It is pointed out that this augmented perception overcomes the problem of GNSS frame loss and achieves high navigation accuracy in grapes.

**FIGURE 1:**
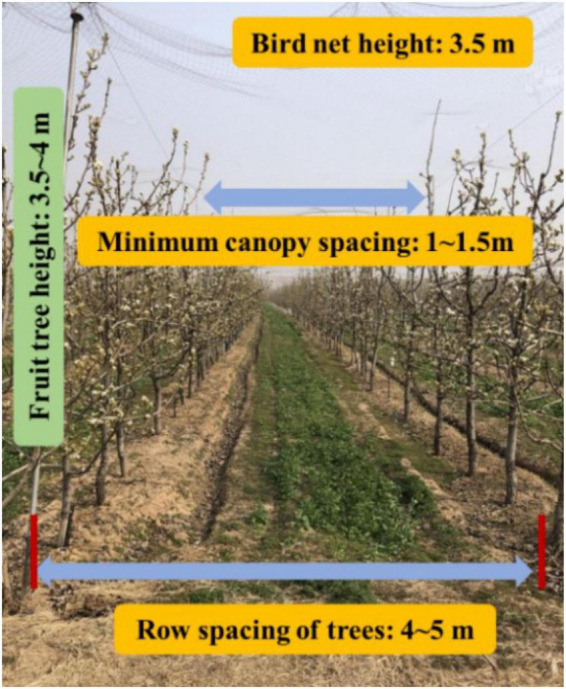
Overview of orchard environment.

Based on the above research, an automatic spray working in orchard is developed in this study using laser radar and millimeter wave radar sensing technology. At the same time, the air spray method is used for pesticides spraying. The crawler chassis and laser radar navigation scheme are adopted according to the standard hedgerow orchard planting mode (as shown in Figure 1). Finally, the full autonomous spray operation is realized in the orchard environment. The main contributions of this work are summarized as follows: (1) An orchard navigation strategy based on laser radar is proposed, and at the same time, combined with ultrasonic radar, the accurate perception of obstacles and high-precision planning of navigation route are realized. (2) An air spray device is developed to realize the twice atomization of liquid medicine and improve the penetration rate of droplets.

The rest of this article is organized as follows: section “Design of hardware system” introduces the hardware part of the system, including the chassis, sensor module, spray system, and other core modules; section “Design of software system” proposes the environment perception and navigation based on lidar, as well as the control strategy; section “Experiments and discussions” discusses the experiments of obstacle avoidance and navigation accuracy under unfair conditions, also the preliminary spray experiment; and section “Conclusion” summarizes the full study.

## Design of hardware system

**FIGURE 2:**
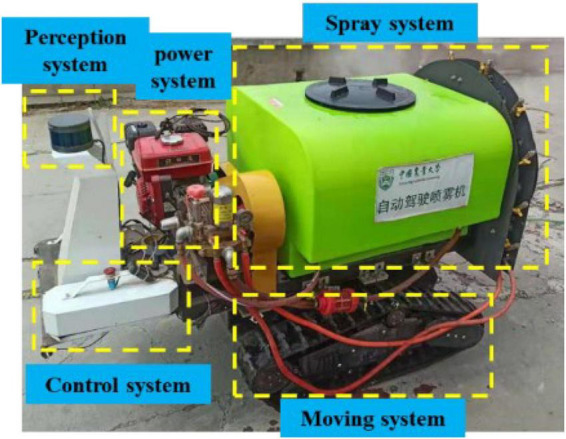
Hardware module of orchard autonomous navigation spray system.

**FIGURE 3:**
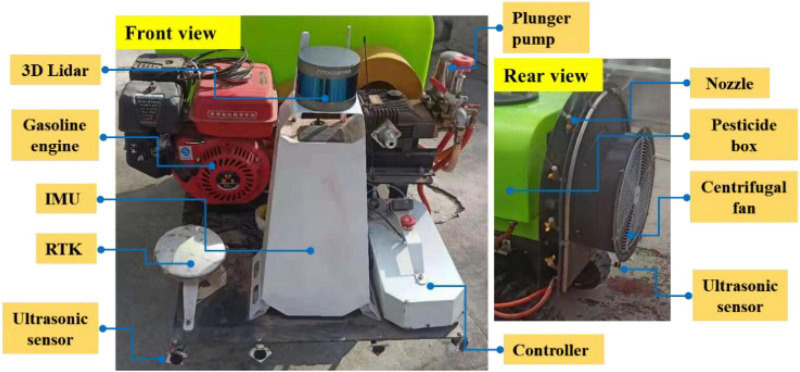
The main hardware distribution of the orchard autonomous navigation spray system.

As shown in Figure 2, the electric air-driven crawler sprayer is composed of power unit, traveling system, transmission device, control device, pneumatic system, spray device, etc. The main components of the electric air-driven crawler sprayer shown in Figure 3 are remote control track chassis, frame, 200-L medicine tank, 48-V Servo electrode, centrifugal fan, water ring, 170-F gasoline engine and centrifugal pulley. The chassis and spray device are powered by different units, respectively. The track chassis is electric, and the 170-F gasoline engine is used as the power unit for the spray device to transmit the power to the centrifugal fan at the rear to ensure sufficient power. Actions such as forward, turning, and moving backward of the machine are realized by the electric part driving the left and right gear motors. The pump pressure of the Model 25A plunger pump can be adjusted by the pressure valve preset, which adopts centrifugal belt installation installed on the output shaft of 170-F gasoline engine, and applies belt transmission between the fan pulley and the plunger pump pulley to form a three-axis linkage. When the output speed of the gasoline engine exceeds 600 rpm/min, the pulley of the walking device begins to work. The fan rotation and the power of the plunger pump are provided by the transmission of the gasoline engine, and the installation height of the plunger pump can be adjusted according to the demand during the actual working process to fully provide tension to the power transmission belt.

### Chassis drive

**FIGURE 4:**
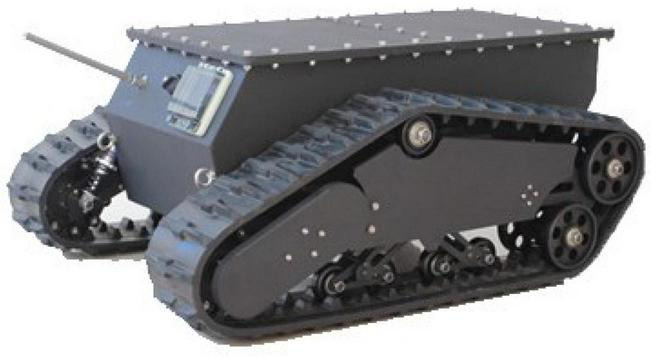
Structure diagram of crawler chassis diagram.

Considering that the platform is mainly used for spray operation in hedgerow orchards, the mechanical part of the navigation robot hardware platform is correspondingly improved. Therefore, the chassis needs to be equipped with a medicine box and spray system weighing about 250 kg. Given that the liquid medicine will shake with the movement of the platform during the operation and the situation of the muddy road, as shown in the Figure 4, the medicine box is embedded into the chassis for obtaining a low-center of gravity-tracked chassis structure. The chassis design parameters are shown in Table 2.

**TABLE 2:** Chassis hardware structure parameters.

Index	Load (kg)	Width (m)	Length (m)
Parameter	250	1.2	1.2
**Index**	**Ground clearance (mm)**	**Turning radius (m)**	**Top speed km/h**
Parameter	150	<7	≥4

### Sensors and information processing modules

**FIGURE 5:**
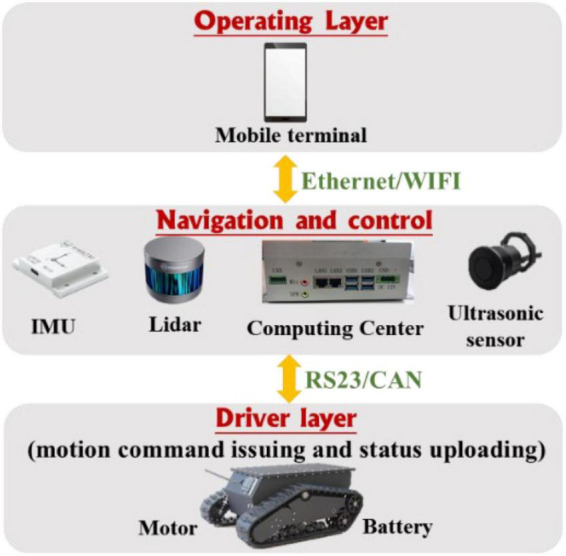
Sensor and information processing module information transmission process.

The sensor module, processor framework, and communication transmission process are shown in Figure 5, where it can be observed that the core of the sensor module is the lidar sensor and the sprayer is equipped with RS-LiDAR-16, i.e., RoboSense^1^ 16-wire laser radar. The RS-LiDAR-16 emits and receives high-frequency laser beams through 16 groups of built-in laser components, and carries out real-time 3D imaging through 360° rotation. The measurement distance can reach 150 m, the accuracy is within ±2 cm, and 300,000 points clouds can be formed per second on average. The vertical angle measurement is 15–15° for ensuring the real-time perception of the environment in the orchard. Also, N100 IMU of Wheeltec Company^2^ is applied to the inertial measurement unit (IMU) with a three-axis accelerometer, a three-axis gyroscope, and a three-axis magnetometer, among which, the accelerometer resolution is less than 0.5 mg, and the range is ±16 g; the gyroscope resolution is less than 0.02°/s; and the range is ±2,000°/s; the magnetometer resolution is 1.5 mg, and the range is ±4,900. The N100 IMU can meet the effective output of inertia parameters such as attitude angle and velocity in orchard environments. The model used by the ultrasonic obstacle avoidance sensor is DYP-A19-V1.0 (Best Sensor)^3^, whose measuring range is 28–450 cm, with an accuracy of ±(1 + 0.3% of the current ranging). The CPU of the control host is i7 4700M, equipped with 8-G memory and 128-G storage for realizing the solution of sensor data, information storage, and output.

### Spray system module

Orchard electric air-driven crawler sprayer can satisfy the requirements for modern orchard plant protection spray operation, and the spray unit is powered by a diaphragm pump. The sprayer follows two atomization processes. First, the liquid medicine is extracted from the medicine box and atomized once through the spray system, when the diaphragm pump is the power source. Then, the liquid medicine passes through the infusion tube and is transported to each nozzle at the ring baffle, and the high-pressure air flow is generated by the centrifugal fan to produce a second atomization of the droplets.

**FIGURE 6:**
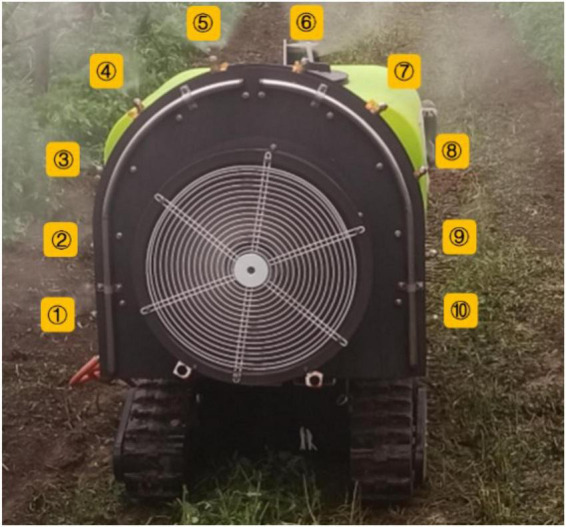
Sprinkler distribution map.

As shown in Figure 6, the spray device of the orchard electric air-driven crawler sprayer is mainly composed of a centrifugal fan, an arc-shaped aqueduct on both sides of the tail and nozzles. Ten spray nozzles are arranged in a circle along both sides at the end of the machine, and five nozzles are evenly arranged on each pipe ring. Each nozzle is equipped with a switch that can be adjusted independently, and the nozzle angle can be adjusted as well; from bottom to top on the left are nozzles No. 1, 2, 3, 4, and 5, respectively, and the nozzles are symmetrically distributed both on the right and the left. The detailed parameters are shown in Table 3. The application of a segmented water ring can ensure that the pressure of each sprinkler is basically the same, and the direction and angle of the nozzle can be adjusted according to the actual growth of the fruit trees in the pear orchard during the spray operation.

**TABLE 3:** List of nozzle parameters from No. 1 to 5.

Nozzle serial number	1	2	3	4	5
Spray pitch (cm)	8	8	8	8	8
Sprinkler angle (°)	0	18	36	54	72
Sprinkler height (cm)	30	46	60	75	88
Wind velocity (m/s)	20	20	20	20	20
Spray volume (L/min)	1.7	1.7	1.7	1.7	1.7

1, 2, 3, 4, and 5 represent the nozzle numbers of the nozzles of the orchard electric air-driven crawler sprayer.

## Design of software system

**FIGURE 7:**
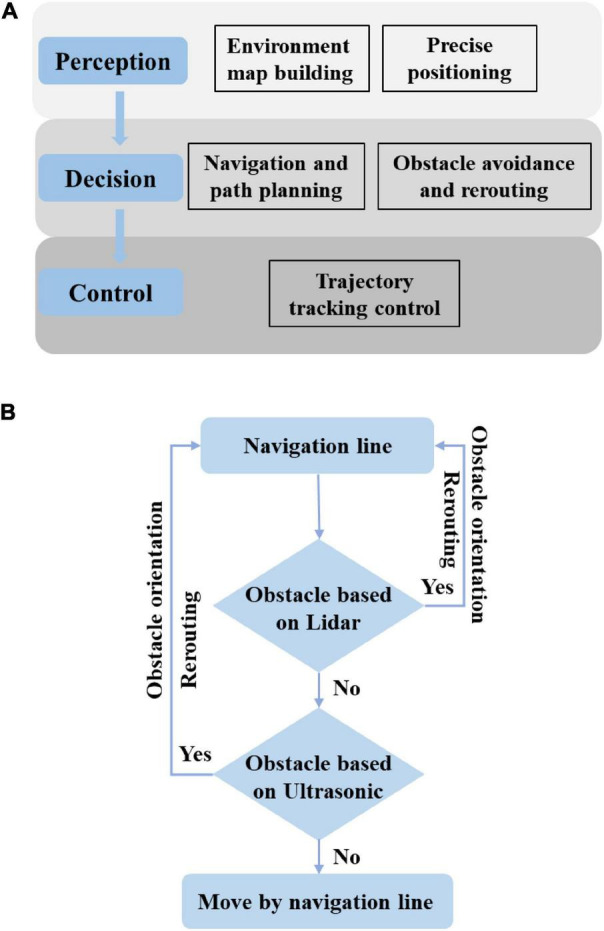
Software block diagram of unmanned spray truck based on “perception decision control”. **(A)** Relationship between three layers. **(B)** Obstacle avoidance fusion output based on decision-level fusion.

The navigation system is provided with the function of switching between manual control and autonomous driving, so that the user can remotely control the chassis to the orchard before operation and switch to the autonomous navigation mode after the navigation task is planned during operation, thereby realizing full autonomous operation. After operation, the user can remotely control the chassis out of the orchard, and handle emergencies by remote control at the same time. As shown in Figure 7A, the overall system is divided into three parts, i.e., perception, decision, and control. First, the local map is constructed based on lidar; then, the operation path is planned in accordance with the constructed map and finally, the planned path is transmitted to the trajectory controller for the unmanned vehicle traveling according to the preset trajectory. At the same time, it can realize automatic obstacle avoidance, including obstacle detection, type recognition, and selective bypassing of static and dynamic obstacles, which are also involved in the perception layer and decision-making layer. As shown in Figure 7B, two sensors based on ultrasonic radar and laser radar are used for obstacle perception. Given that the longitudinal sensing range of laser radar is only 30, it is difficult to perceive ground obstacles. The ultrasonic radar located under the vehicle is used for obstacle fusion sensing. The two sensing strategies are fused based on the decision-making layer, and the weighted decision-making method is adopted. Each laser radar and ultrasonic radar have 50% weight. When the proportion of perceived obstacles is greater than or equal to 50%, the path needs to be replanned, that is, when any sensor senses an obstacle, the path needs replanning. The overall software is secondary developed based on Autopilot Kit.^4^

### Environment perception and navigation based on laser radar

**FIGURE 8:**
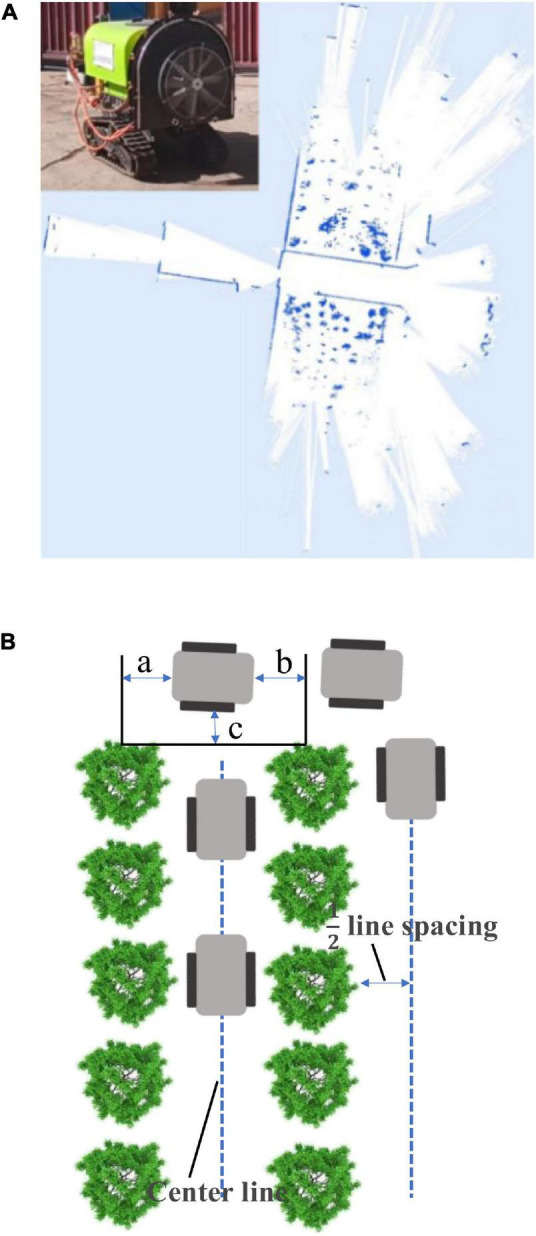
Construction of local map based on RS-LiDAR-16. **(A)** Local map acquisition. **(B)** Schematic diagram of navigation line.

As shown in Figure 8A, the automatic tracking navigation can be realized by constructing a local map through laser radar. The navigation system detects the fruit trees and takes the two lines of center lines as the traveling track to carry out the traveling operation when the spray truck travels in the orchard, and can automatically complete the turning and move to the next row of operation when reaching the ground, as shown in Figure 8B. The navigation rules are as follows: The centerline is taken as the travel track when traveling in two rows; the trajectory is determined by a distance of one-half row when there are fruit trees on only one side; when the spray truck reaches the end-member of field, *a* = *b* = 1/2 line spacing (determined at the center), and C is not less than the safe distance. The next center position is determined as the spray truck moves on to the next row and turns to move on to the next row of work.

**FIGURE 9:**
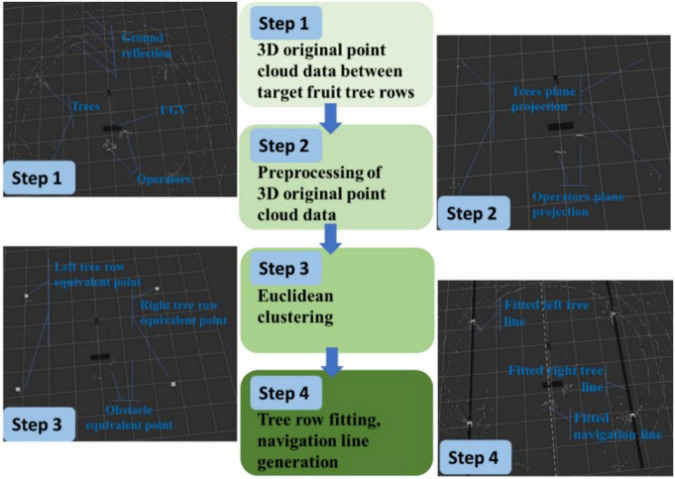
Navigation line extraction rules among fruit trees.

As shown in Figure 9, the navigation line extraction rules of the spray truck between fruit trees are divided into four steps: Step 1: Data collecting of the 3D original point cloud data between the rows of the target orchard; Step 2: Data preprocessing (clipped and dimensionality reduction) of the 3D original point cloud data; Step 3: European clustering; and Step 4: Tree row fitting and the navigation line generation. Because the extreme weather, such as rain, fog, and high temperature, is not suitable for spray operation, this study does not consider extreme weather navigation line extraction.

The original point cloud data of the 3D space obtained between the rows of the target orchard are collected, as shown in Figure 9 (Step 1), where the block is a mobile robot model; other white point clouds are the relative positions of the objects in the 3D scene; the coordinate origin, such as the point clouds on the left and right sides of the mobile robot are fruit trees; those in front are ground reflections; and those in the back are the mobile robot operators. Point clouds have a 360° horizontal full coverage and a vertical coverage from the ground to above the fruit trees. The RS-LiDAR-16 laser radar adopted in this study is a 16-wire laser radar with a horizontal field of view of 360°, a vertical field of view of 30° (±15°), and a maximum detection distance of 200 m.

In Step 2, the preprocessing of the 3D original point cloud data is mainly completed *via* clipping and dimensionality reduction. The 3D clipping mainly involves data clipping based on the *x*-, *y*-, and *z*- axes, and the specific clipping process is as follows: The clipping threshold of *xyz* is set according to the plant spacing, row spacing, and the average net trunk length of fruit trees; that of *x*-axis is at least three times of single row spacing; that of *y*-axis is at least 1.5 times of single row spacing; and that of *z*-axis does not exceed the average net trunk length. The clipping purpose is to select a certain visual range, reduce the amount of data, and improve the processing speed.

The dimensionality reduction of the cropped data mainly aims to project the cropped 3D data into a given 3D space plane (*x* = 0, *y* = 0, *z* = 1), thereby realizing the dimensionality data reduction from 3D to 2D, and simplifying the geometric problem, as shown in Figure 9 (Step 2). The left and right points in the figure are the projection of the trunk in the plane (*x* = 0, *y* = 0, *z* = 0), while the middle point denotes the projection of the operator in the plane (*x* = 0, *y* = 0, *z* = 1).

Euclidean clustering method is used in Step 3 for clustering the effective points after search. The midpoint of each category of the data is calculated to replace the corresponding category, and equivalent points of two left tree row, the obstacle, and the right tree row are all marked as shown in Figure 9 (Step 3).

Finally, the left tree row straight line and the right tree row straight line are made to fit using the least square method in Step 4. The left tree row equation and the right tree row equation are obtained as shown in Figure 9 (Step 4), and the fitted left and right tree row lines are marked, respectively. Finally, the center line of the left and right tree rows is adopted for calculating the navigation line, and the fitted navigation line is also marked in the map.

### Control strategy

**FIGURE 10:**
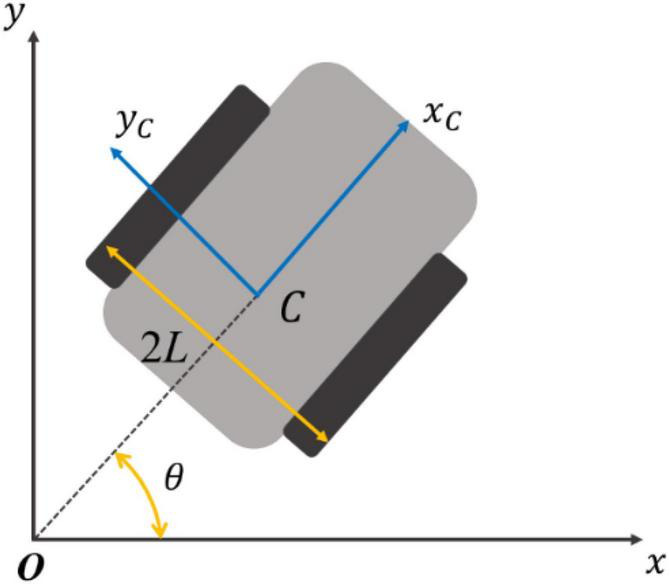
Definition of crawler coordinate system.

The hereby designed tracked vehicle controls the speed and direction of the driving wheels on both sides for an accurate tracking of the desired trajectory as shown in Figure 10, where _*Oxy*_ is the geodetic coordinate system; _*Cx_c y_c*_ is the tracked vehicle coordinate system;_*C*_ is the coincidence point between the geometric center and the centroid of the tracked vehicle; and _2L_ is the center distance of the tracked vehicle.

The status quantity of tracked vehicle is _*q=(x,y,*θ)_^T, where^_(*x,y*)_ represents the position of tracked vehicle, and the kinematic model of tracked vehicle is calculated as:


(1) $$\left[\begin{array}{c} \dot{x}\\ \dot{y}\\ \dot{\theta } \end{array}\right]=\left[\begin{array}{cc} \begin{array}{c} \cos\theta \\ \sin\theta \\ 0 \end{array} & \begin{array}{c} 0\\ 0\\ 1 \end{array} \end{array}\right]\,\left[\begin{array}{c} v\\ \omega \end{array}\right]$$

where _*v*_ is the vehicle speed; _ω_ is the angular velocity of vehicle centroid; _*vR*_ is the right track speed; and _*vL*_ is the left track speed.

It can be seen that _*u*=[*v,ω*]_^T^ is the control quantity of crawler, when the track vehicle control problem turns into tracking the track vehicle reference trajectory by finding a suitable control quantity under the condition of given initial state information and speed information. Deriving the kinematic model further, it can be obtained as follows:


(2) $$\left[\begin{array}{c} v\\ \omega \end{array}\right]=\frac{1}{2}\,\left[\begin{array}{cc} 1 & 1\\ -\frac{1}{L} & \frac{1}{L} \end{array}\right]\,\left[\begin{array}{c} v_{L}\\ v_{R} \end{array}\right]$$

**FIGURE 11:**
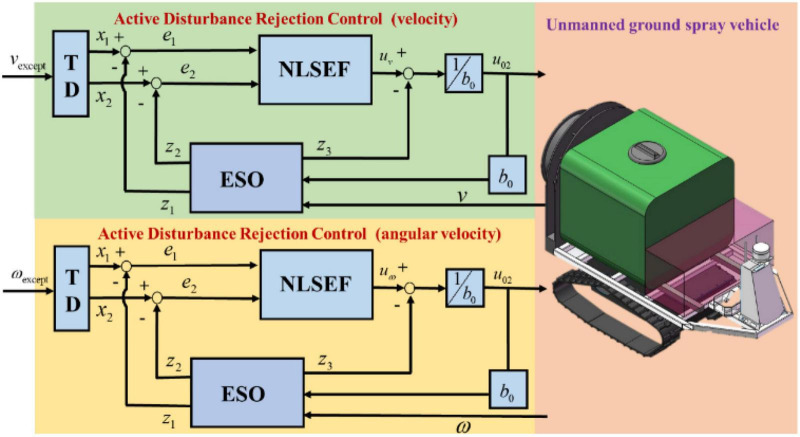
System control block diagram based on ADRC (ESO, extended state observer; TD, tracking differentiator; NLSEF, non-linear state error feedback).

The control of speed and steering angle is particularly important during the operation of tracked plant protection vehicle. Assuming the nozzle sprays liquid medicine at a constant flow rate, the unmanned vehicle is required to travel at a constant speed. Whether the unmanned vehicle needs human participation in the control at the boundary turn of the plot is determined by whether the steering angle can be accurately controlled. In addition, during the operation of the plant protection unmanned vehicle, the total mass of the vehicle will be reduced with the spraying of liquid medicine, and the shaking of liquid medicine in the medicine box, air resistance, non-linear friction, and the unmodeled part of the system will cause multi-source and unknown interference to the agricultural unmanned vehicle. For solving these problems, the active disturbance rejection control (ADRC) control strategy is adopted for the controller of the plant protection unmanned vehicle. The control system is designed based on the discussions in Wang S. et al. (2022), as shown in Figure 11, with the following two ADRC controllers involved: One receives the desired speed and outputs the speed control quantity while the other receives the desired angular speed and outputs the angular speed control quantity. At the same time, the tracked vehicle transmits the actual speed and angular speed to the controller.

## Experiments and discussions

**FIGURE 12:**
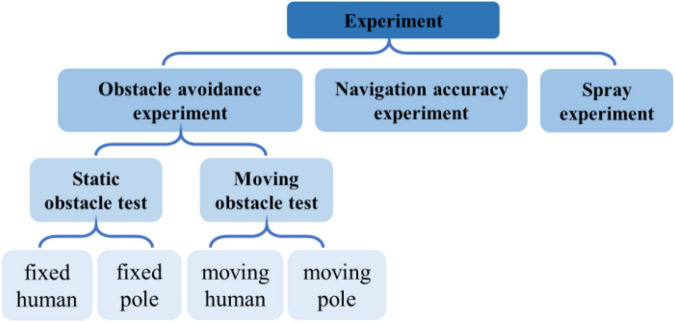
Experimental flow chart.

In this section, we discuss on several groups of obstacle avoidance experiments, such as navigation accuracy experiments and fog drop coverage tests that are designed; also discussed are the obstacle avoidance experiments that include static obstacle experiments and dynamic obstacle experiments. The specific experimental process is shown in Figure 12.

### Obstacle avoidance performance test

#### Static obstacle test

##### Fixed human obstacles

**FIGURE 13:**
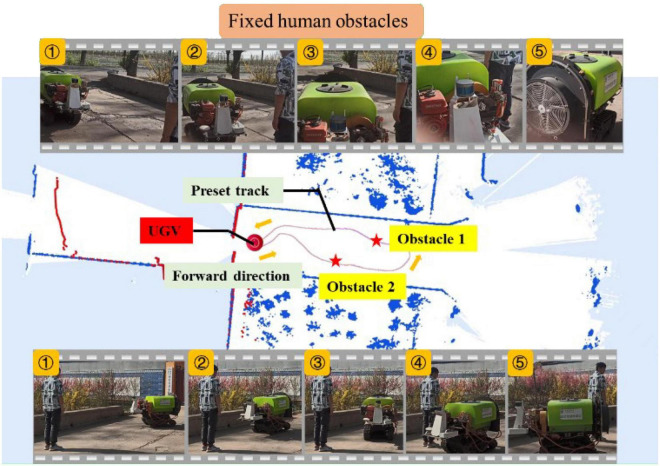
Experiment of fixed human obstacle.

In this experiment, the size of the selected work area was about 10 m × 15 m. First, the closed-loop track was formed by manually manipulating the UGV as shown in Figure 13, where the orange arrow indicates the direction of travel. During manual operation, the lidar combined with the IMU data would map the working area, and then switch to the automatic mode, when the UGV would return to the starting point of the manual operation, and follow the manual operation track automatically for trajectory tracking control. At this time, two fixed persons were set as obstacles in the track as shown in the figure. The upper part of the figure depicts the key five-frame pictures of the UGV avoiding obstacle 1, where it can be observed that the UGV could identify and avoid obstacles well: the UGV in the test started braking at a distance of about 0.5 m from the obstacle, bypassed the person from the right, and quickly returned to the set track to continue driving. The lower part of the figure describes the key five-frame pictures of the UGV avoiding obstacle 2. Similar to facing obstacle 1, the UGV started braking at a distance of about 0.5 m from the person, turned left to avoid the person, and finally returned to the preset track. In the fixed-person obstacle experiment, the height of the person was about 1.8 m and the width was about 0.5 m. The UGV could avoid obstacles well and return to the preset track as expected.

##### Fixed pole obstacles

To further test the UGV’s capability of recognizing the size of obstacles, resin tubes and wooden strips were selected for obstacle avoidance experiments. The height of the resin tube was about 1.5 m and the diameter was about 0.05 m; the height of the wood strip was about 1.5 m and the diameter was about 0.04 m. The same terrain (10 m × 15 m) was selected for two groups of experiments whose preset trajectories were different. The placement position of obstacles varied as well.

**FIGURE 14:**
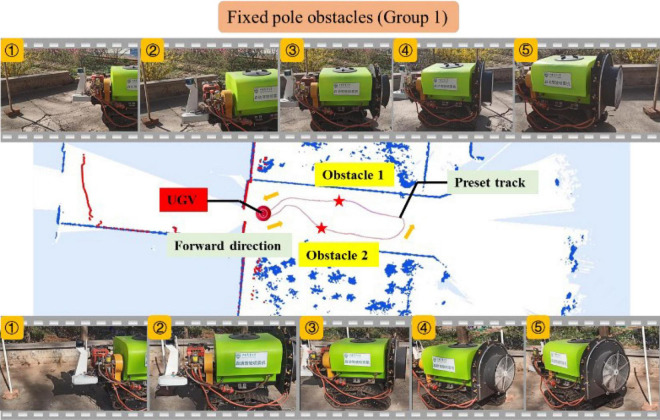
Experiment of fixed pole obstacle (Group 1).

As shown in Figure 14, the upper part of the figure displays the key five-frame pictures of the UGV avoiding obstacle 1 when the wooden strip was used as an obstacle. It can be seen from the image of Frame 1 in the figure that the unmanned vehicle recognized the obstacle and braked sharply when it was about 0.5 m away from the wooden strip. Frames 1, 2, 3, and 4 show that the UGV started to bypass the obstacle at this time, while Frame 5 presents that the UGV had completed the detour process and was returning to the preset track. The lower part of the figure shows the five key frames of the UGV bypassing obstacle 2 of the resin tube. Similar to the wooden strip experiment, the UGV stopped about 0.5 m away from the obstacle, continued to bypass the obstacle, and returned to the preset track as expected.

**FIGURE 15:**
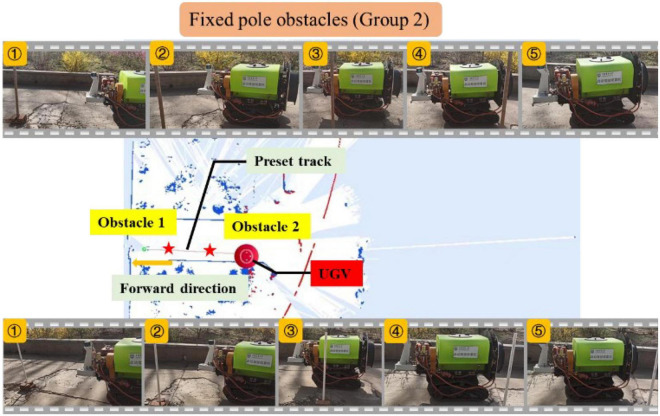
Experiment of fixed pole obstacle (Group 2).

The second set of experiment was also carried out, and the preset track was redesigned in the experiment, as shown in Figure 15. The upper and lower parts of the figure still show the five key frames of the UGV avoiding obstacles when the wood strips and resin pipes were used as obstacles, respectively. Similar to the previous set of experimental results, the unmanned vehicle stopped quickly at a distance of 0.5 m from the obstacle, avoided the obstacle by detouring, and finally returned to the preset track. Replacing the curve preset track with the straight-line preset track can better display the process of UGV bypassing obstacles and returning to the preset track. Besides, the UGV deviated from the preset track by about 0.3–0.5 m while bypassing the obstacles.

#### Moving obstacle test

##### Moving human obstacles

**FIGURE 16:**
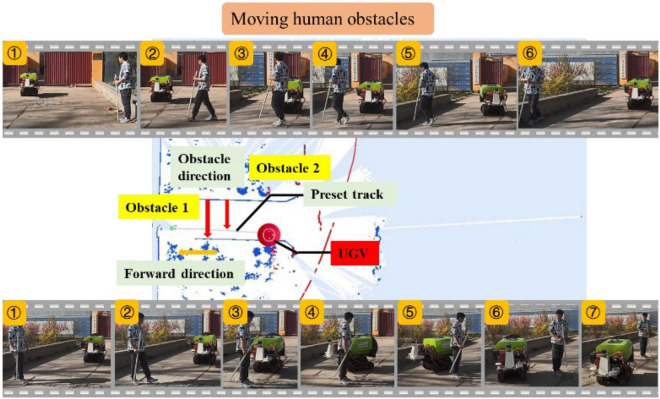
Experiment of moving person obstacle.

To further test the obstacle avoidance performance of the UGV, dynamic obstacles were set in this section. As shown in Figure 16, the travel track of the UGV, and two groups of moving person obstacles were all set in advance during the travel process. The UGV followed the red arrow to travel to the other side when passing the moving person obstacle 1, and followed the red arrow to start and stop on the track when passing the moving person obstacle 2. The upper part of Figure 16 depicts the six key frames of the UGV avoiding obstacle 1 with a moving person as the obstacle. It can be seen from the image of Frame 1 that the UGV was traveling according to the preset track at this time, and in Frame 2, the person had started to set off, and the tracks of human and UGV would coincide. In Frames 3 and 4, the UGV completely coincided with the person’s movement track, when the UGV started to stop suddenly and tried to detour. The person continued to move. It can be seen in Frame 5 that the UGV had an obvious detour, and the heading angle was deflected, but the moving person had left the preset track at this time. In Frame 6, the person had completely left the preset track, the UGV returned to the preset track and continued to move forward.

The lower part of Figure 16 depicts the seven key frames of the UGV avoiding obstacle 2 with a moving person acting as the obstacle. It can be seen from Frame 1 that the UGV was traveling according to the preset track at this time, and in Frame 2, the person had started to set off, and the tracks of human and the UGV would coincide. In Frame 3, the person had stopped on the UGV preset track, and the UGV started to stop suddenly. Frames 4, 5, and 6 show that the UGV bypassed the stopped person and started to approach the preset track. Frame 7 shows that the UGV had completed the obstacle avoidance of the moving person, completely returned to the preset track, and continued to travel. The experiments of two different modes of moving people as obstacles show that the UGV also had good detection and obstacle avoidance performance for unknown moving obstacles. Especially for the sudden obstacles, it could quickly brake and make evasive actions, and return to the preset track.

##### Moving pole obstacles

**FIGURE 17:**
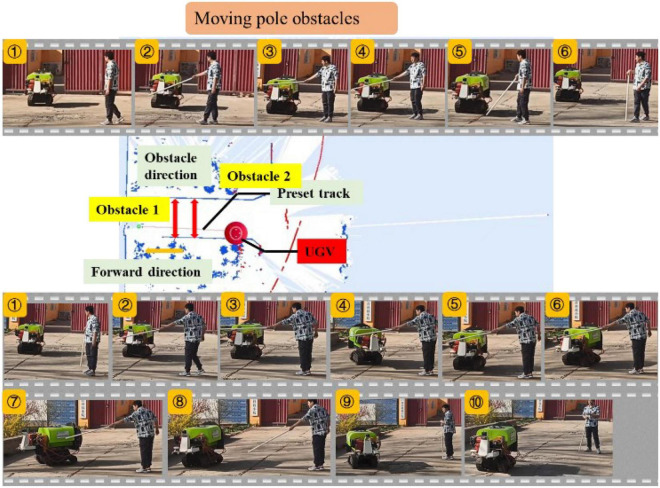
Experiment of moving pole obstacle.

To further test the obstacle avoidance ability of UGV for small-sized and moving obstacles, the experiment of hand-held moving resin rods as obstacles was carried out. As shown in Figure 17, two groups of moving resin rods were set as obstacles during the UGV’s traveling process, the hand-held moving rod was placed in front of the UGV for 5 s and then withdrawn when the UGV passed the moving resin rod 1, and when the UGV passed the moving resin rod 2, the hand-held moving rod had been placed in front of the UGV for 30 s.

The upper part of Figure 17 describes the key six-frame picture of the UGV bypassing obstacle 1 with the moving rod acting as the obstacle. It can be seen from Frame 1 in Figure 16 that the UGV traveled according to the preset trajectory. In Frames 1 and 2, the resin rod was picked up and placed in front of the UGV, when the UGV started to brake and make an emergency stop to avoid obstacles. The emergency stop was maintained until Frames 4 and 5. In Frame 6, the moving resin rods were evacuated and the UGV started to move on.

The lower part of Figure 17 shows the 10 key frames of the UGV bypassing obstacle 2 with the moving rod acting as the obstacle. It can be seen from Frames 1, 2, and 3 that the UGV traveled according to the preset trajectory, when the resin rod was picked up and placed in front of the UGV, and the UGV started to brake and make an emergency stop to avoid obstacles. Compared with the previous groups of experiments, the moving rod was placed in front of the UGV for a longer time, about 30 s. It can be seen from Frame 5 that the UGV started to deflect the heading angle to the left, trying to avoid obstacles, but if it moved forward to the left, human would appear as obstacles. In Frames 5 and 6, the UGV turned around the yaw angle and deflected to the right, and began to prepare for detour, and then in Frames 7 and 8, the UGV bypassed the obstacle from the side. In Frame 9, the obstacle was successfully avoided and the preset track was approached. In Frame 10, it completely returned to the preset trajectory and continued to move forward to complete the obstacle avoidance process.

In the moving rod experiment, the diameter of the moving rod was only 0.05 m. When the moving rod suddenly appeared in front of the UGV, the UGV detected it, and when the obstacle stayed for a long time, the UGV made a detour. The above groups of experiments verify the real-time detection performance of the designed UGV for small-sized and moving obstacles.

### Navigation accuracy experiment

**FIGURE 18:**
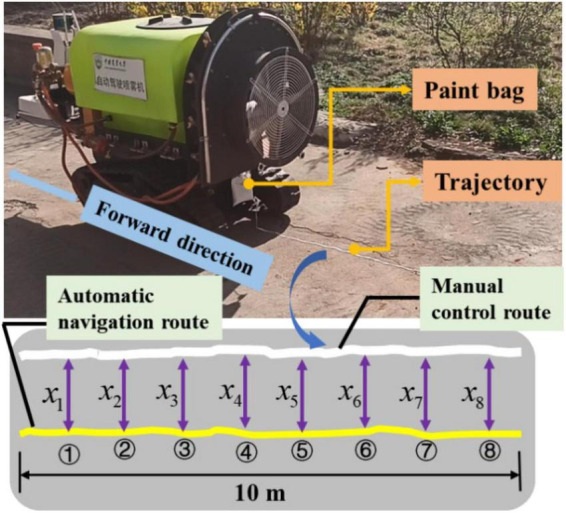
Navigation accuracy test scheme.

To verify the navigation accuracy of the UGV automatic navigation system, the evaluation was performed by designing and measuring the difference between the manual navigation route and the automatic navigation route. As shown in Figure 18, the paint would flow out from the paint bag and form a paint line on the ground for recording the travel track in real time by hanging the paint bag at the tail of UGV under the real environment. The manual control was the white paint line; the automatic navigation was the yellow paint line; the length of the selected track line was about 8 m; and a point was selected every one meter as the measurement point (_*x1,x2,x3,x4 x5,x6,x7,x8*_); and the automatic navigation performance was evaluated by measuring the distance between the yellow paint line and the white paint line. As shown in the formula provided in Eq. (3), the average value was taken as the navigation accuracy of the planned route.


(3) $$\mathrm{Navigation}\,\mathrm{accuracy}=\left|\frac{x_{1}+x_{2}+x_{3}+x_{4}+x_{5}+x_{6}+x_{7}+x_{8}}{8}\right|$$

#### Navigation accuracy experiment (Group 1)

**FIGURE 19:**
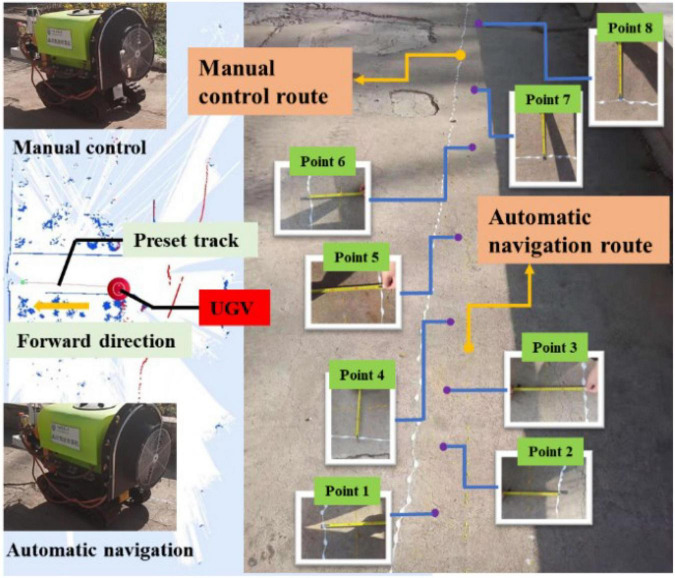
Navigation accuracy experiment (Group 1).

The size of the operation area was about 10 m × 15 m in the first set of navigation accuracy experiment. First, the UGV was manually controlled to form the trajectory shown in Figure 19, when the paint bag hanging at the tail of the UGV was filled with white paint, forming a white paint trajectory line on the ground. Then, the UGV was switched to the automatic mode and returned to the starting point of manual control, and automatically followed the manually operated track for the track tracking control. At this time, the paint bag hanging at the tail of the UGV was yellow paint, forming a yellow paint track line on the ground. The eight selected points were shown in Figure 19, and the measurement results were shown in Table 4. It can be concluded from Table 4 that the error of each point was within 15 cm in the eight selected points, and the average navigation accuracy was 13.625 cm, meeting the navigation accuracy requirements of the UGV working in the orchard.

**TABLE 4:** Navigation accuracy test value of each sampling point (Group 1).

Collection point	Point 1	Point 2	Point 3	Point 4	Point 5
Measured value	12 cm	14 cm	14 cm	15 cm	14 cm
**Collection point**	**Point 6**	**Point 7**	**Point 8**	**Average**	
Measured value	13 cm	14 cm	13 cm	13.625 cm	

#### Navigation accuracy experiment (Group 2)

**FIGURE 20:**
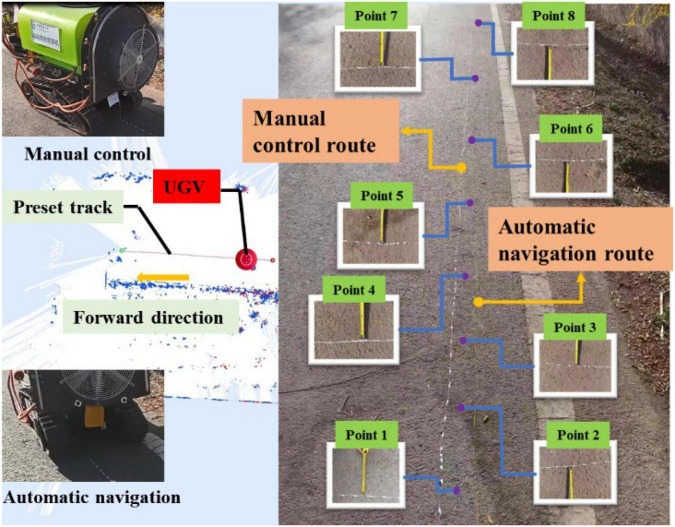
Navigation accuracy experiment (Group 2).

The size of the operation area was about 10 m × 10 m in the second group of navigation accuracy experiment. The UGV was still manually controlled to form a white paint track line on the ground at first, and was then switched to the automatic mode for the tracking control, forming a yellow paint track line on the ground. Similarly, eight points were selected as the sampling points, as shown in Figure 20. The measurement results of each point were shown in Table 5, where it can be observed that the error of each point was still within 15 cm, and the average accuracy was 10.3125 cm, which, compared with the first group of accuracy experiments, was much improved. Given that the test site of the second group was flatter than the first group, there was less shaking of the UGV.

**TABLE 5:** Navigation accuracy test value of each sampling point (Group 2).

Collection point	Point 1	Point 2	Point 3	Point 4	Point 5
Measured value	14 cm	12 cm	11 cm	10 cm	10.5 cm
**Collection point**	**Point 6**	**Point 7**	**Point 8**	**Average**	
Measured value	10 cm	3 cm	12 cm	10.3125 cm	

In the above two groups of accuracy experiments, the error of all sampling points was within 15 cm, and the navigation accuracy was about 10 cm in the relatively flat area, meeting the navigation requirements of the orchard spray system.

### Spray experiment

The standard orchard demonstration area in Xiying Village was selected for the spray test. The specific parameters of the planting mode were as follows: The orchard area was more than 300 acres; the spacing of each row of fruit trees was about 3 m; the spacing between the trees was 1.5 m; the growth height of the fruit trees was 3 m; and the height of the trunk was about 0.5 m. The canopy of fruit trees was of small crown and sparse layer type, with a crown diameter length of 1.5 m, which could be divided into three layers in the vertical direction. The whole tree retained one main branch, while the side branches were less reserved and the branches were simple. The angle with the central trunk was between 60${}^{{}^{\circ}}$ and 80°, and the pruning method resembled the spindle shape.

**FIGURE 21:**
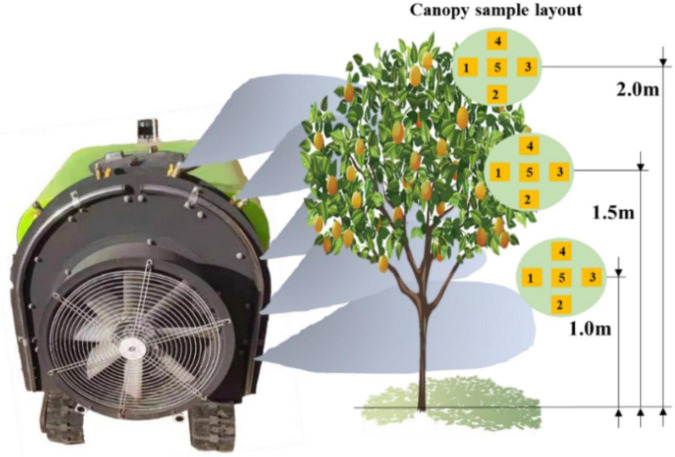
Spray diagram and collection point layout.

Three trees set the five positions of east, south, west, north and middle in the canopy were selected. The first layer was 1 m away from the ground, and every 50 cm from the bottom to the top of the canopy was used as a layer, and then, the second layer and the third layer in the same way. Besides, the water-sensitive paper was fixed on the leaves of the east, south, west, north, and middle with an alligator clip, and three trees were continuously arranged from west to east along the traveling direction of machines and tools. The arrangement of canopy droplet samples was shown in Figure 21. The scanner was used for obtaining the spray landing area of droplets on each piece of water-sensitive paper, and finally obtaining the coverage per unit area as shown in Table 6.

**TABLE 6:** Coverage of each sampling area under different layers.

Sampling point	1	2	3	4	5
Upper layer/100%	75.2	69.5	46.5	59.4	60.3
Middle layer/100%	50.7	33.9	43.3	58.5	45.2
Lower level/100%	79.2	43.5	27.5	56.5	51.4

The coverage rate of the leaves of the canopy was almost no more than 80%, most of them gathering at 50%, when the spray effect was consistent with the growth conditions of the pear trees.

## Conclusion

At this stage, automation technology has been widely transferred to orchard equipment, which has promoted the intelligent development of agricultural equipment. Aiming at the problem of automatic spray in complex and closed orchard environment, a 3D laser lidar orchard map construction strategy is adopted in this study, and at the same time, the air spray is selected to realize two atomization of the liquid medicine and improve the penetration rate of the droplets. The 3D laser lidar can facilitate all-weather orchard operations compared to the characteristics of visual navigation greatly affected by light, which is necessarily important for the large-scale occurrence of diseases and pests, also the urgent need for fast operation time. Millimeter wave radar is selected for obtaining multi-source information of obstacle avoidance, which improves the accuracy of obstacle avoidance. However, the autonomous navigation spray system developed in this study fails to take much account of the spray system, such as variable spray and profiling spray technology. To this end, the precision spray technology will be further explored to achieve independent and accurate pesticide spraying in the orchard environment on the basis of automatic navigation.

## Data availability statement

The original contributions presented in the study are included in the article/Supplementary material, further inquiries can be directed to the corresponding authors.

## Author contributions

SW, JS, PQ, CY, HW, LZ, and WL: conceptualization, methodology, software, validation, formal analysis, investigation, resources, data curation, writing the original draft, reviewing and editing, and visualization. YL and XH: supervision, funding acquisition, and project administration. All authors contributed to the article and approved the submitted version.

## Footnotes

1
https://www.robosense.cn/en

2
http://wheeltec.net/product/html/?144.html

3
http://www.dypsensor.com/en/proData.aspx?cid=142&pid=122

4
https://www.agilex.ai/solution/5?lang=zh-cn
